# COVID-19 Case Rates in Transitional Kindergarten Through Grade 12 Schools and in the Community — Los Angeles County, California, September 2020–March 2021

**DOI:** 10.15585/mmwr.mm7035e3

**Published:** 2021-09-03

**Authors:** Sherry Yin, Kaitlin Barnes, Rebecca Fisher, Dawn Terashita, Andrea A. Kim

**Affiliations:** ^1^Los Angeles County Department of Public Health, California; ^2^Los Angeles County Office of Education, California.

In-person instruction during the COVID-19 pandemic concerns educators, unions, parents, students, and public health officials as they plan to create a safe and supportive learning environment for children and adolescents (*1*). Los Angeles County (LAC), the nation’s largest county, has an estimated population of 10 million, including 1.7 million children and adolescents aged 5–17 years (*2*). LAC school districts moved to remote learning for some or all students in transitional kindergarten* through grade 12 (TK–12) schools during the 2020–21 school year (*3*). Schools that provided in-person instruction were required by LAC Health Officer orders to implement prevention measures such as symptom screening, masking, physical distancing, cohorting, and contact tracing (*4*). This analysis compares COVID-19 case rates in TK–12 schools among students and staff members who attended school in person with LAC case rates during September 2020–March 2021.

LAC schools are required to report all laboratory-confirmed COVID-19 cases in persons who were on campus during their incubation or infectious period to the LAC Department of Public Health (DPH).^†^ School-associated cases were defined as cases among students and staff members who were on campus for any length of time from 14 days before symptom onset or testing (whichever was earlier) until isolation. Cases among students and staff members who participated exclusively in online learning or worked remotely were not considered school-associated cases. DPH and the LAC Office of Education also collected information from the county’s 80 public school districts on the estimated number of students and staff members routinely on campus each month during September 2020–March 2021. Monthly attendance was prorated based on when schools opened and ranged from 2,738 to 62,369 students and 36,862 to 45,757 staff members. Student and staff member case rates were calculated using the number of school-associated cases reported to DPH, assigned to a month by episode date, and divided by monthly attendance. Community case rates among children and adolescents aged 5–17 years and adults aged 18–79 years were calculated using the number of reported cases in LAC divided by the 2019 county population.^§^ Standard errors were calculated for school-associated rates. Analyses were conducted using SAS (version 9.4; SAS Institute). This public health surveillance activity was approved by DPH.

During September 1, 2020–March 31, 2021, a total of 463 school-associated cases were reported among students attending public TK–12 schools in person and 3,927 among staff members working on-site. During the same period, 105,577 cases among children and adolescents aged 5–17 years and 771,409 cases among adults aged 18–79 years were reported in LAC. School-associated case rates remained low among students, ranging from 110 per 100,000 in September to 859 in December 2020 (Figure). Case rates among all children and adolescents aged 5–17 years in the county were higher during most of the period, ranging from 167 per 100,000 in September to 2,938 in December 2020. School-associated case rates among staff members were lowest in September 2020 (125 per 100,000), peaked in December 2020 (4,109), and fell sharply through March 2021 (188). These rates reflected the trend among all adults aged 18–79 years in the county (319 per 100,000 in September 2020; 4,624 in December 2020; and 181 in March 2021) but were lower for most of the period. As total cases fell sharply in February, rates across the four groups declined to similar levels by March 2021.

**FIGURE:**
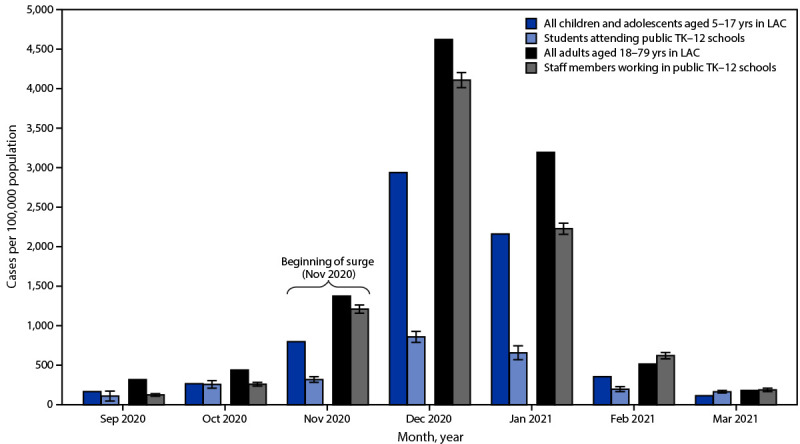
COVID-19 case rates* among children, adolescents, and adults^†^ in transitional kindergarten through grade 12 schools and in the community, by month — Los Angeles County, California, September 2020–March 2021 **Abbreviations**: LAC = Los Angeles County; TK–12 = transitional kindergarten through grade 12. * New cases per month per 100,000 persons; standard error bars shown for school case rates. ^†^ Adult staff members comprise all school employees and associated workers on campus, including teachers, nurses, public safety officers, administrative staff members, campus aides, food service workers, custodians, and transportation staff members.

The findings in this report are subject to at least three limitations. First, these findings from one county should be interpreted with caution and are not necessarily generalizable to other areas. Second, the analysis did not include the entire school year because estimates of students and staff members on campus were collected only through March 2021. Finally, because of limited available information about the population on campus, rates were unadjusted and did not examine potential differences in demographic and socioeconomic characteristics by in-person status. However, sensitivity analysis showed similar trends across LAC’s eight Service Planning Areas.

The findings suggest that implementing recommended prevention measures might protect children, adolescents, and adults from COVID-19 in TK–12 schools. The level of protection appears to be higher in children and adolescents than in adults, which is promising for children aged <12 years because no COVID-19 vaccine is currently authorized for this age group. In schools with safety protocols in place for prevention and containment, case rates in children and adolescents were 3.4 times lower during the winter peak compared with rates in the community. This analysis reflects transmission patterns before the more transmissible SARS-CoV-2 B.1.617.2 (Delta) variant became predominant in the United States. A multipronged prevention strategy, including masking, physical distancing, testing, and most recently vaccination of children and adolescents aged ≥12 years, will remain critical to reducing transmission as more students return to the classroom (*5*). These findings from a large and diverse county present preliminary evidence that schools provided a relatively safe environment during the 2020–21 school year.

## Footnotes

* Transitional kindergarten is a public school program serving to bridge preschool and kindergarten.

† During the observation period, school districts recommended diagnostic testing for students and staff members to identify persons with active infection based on symptoms and exposure. In addition to mandatory laboratory or provider reporting of positive COVID-19 nucleic acid amplification test (NAAT) and antigen test results, schools also notified DPH of cases occurring on campus. Schools relied on staff members, students, parents and guardians, and testing programs where available to inform them of cases and worked with DPH to identify and provide testing for close contacts on campus. The Los Angeles Unified School District (largest of the 80 school districts in the county) implemented a screening testing program using NAATs for staff members and students on campus beginning September 2020 and reported all positive results to DPH.

§ Cities of Long Beach and Pasadena were not included because they have their own health departments separate from LAC DPH. The adult age range reflects the age range of staff members working at TK–12 schools in LAC during the analysis period.
